# Epidemiology of hemorrhagic fever with renal syndrome in Tai’an area

**DOI:** 10.1038/s41598-021-91029-1

**Published:** 2021-07-05

**Authors:** XiuJuan Bi, Shuying Yi, Aihua Zhang, Zhenghua Zhao, Yunqiang Liu, Chao Zhang, Zhen Ye

**Affiliations:** 1 Department of Health Management, The First Affiliated Hospital, Shandong Provincial Qianfoshan Hospital, Shandong Engineering Laboratory for Health Management, Shandong Medicine and Health Key Laboratory of Laboratory Medicine, Shandong First Medical University, Jinan, Shandong People’s Republic of China; 2 Department of Biochemistry and Molecular Biology, Basic Medical College, Shandong First Medical University, Shandong Academy of Medical Sciences, Tai’an, Shandong People’s Republic of China; 3 Department of Infectious Disease, Tai’an Center for Disease Control and Prevention, Tai’an, Shandong People’s Republic of China; 4 Department of Infectious Disease, Tai’an Central Hospital, Tai’an, Shandong People’s Republic of China; 5 College of Information Science and Engineering, College of Information Science and Engineering, Shandong Agricultural University, Tai’an, Shandong People’s Republic of China; 6 Science and Technology Innovation Center, Shandong First Medical University, 6699 Qingdao Road, Jinan, 250117 Shandong People’s Republic of China

**Keywords:** Infectious diseases, Viral infection

## Abstract

Hemorrhagic fever with renal syndrome (HFRS), a serious threat to human health, is mainly transmitted by rodents in Eurasia. The risk of disease differs according to sex, age, and occupation. Further, temperature and rainfall have some lagging effects on the occurrence of the disease. The quantitative data for these factors in the Tai’an region of China are still unknown. We used a forest map to calculate the risk of HFRS in different populations and used four different mathematical models to explain the relationship between time factors, meteorological factors, and the disease. The results showed that compared with the whole population, the relative risk in rural medical staff and farmers was 5.05 and 2.00, respectively (*p* < 0.05). Joinpoint models showed that the number of cases decreased by 33.32% per year from 2005 to 2008 (*p* < 0.05). The generalized additive model showed that air temperature was positively correlated with disease risk from January to June, and that relative humidity was negatively correlated with risk from July to December. From January to June, with an increase in temperature, after 15 lags, the cumulative risk of disease increased at low temperatures. From July to December, the cumulative risk decreased with an increase in the relative humidity. Rural medical staff, farmers, men, and middle-aged individuals were at a high risk of HFRS. Moreover, air temperature and relative humidity are important factors that affect disease occurrence. These associations show lagged and differing effects according to the season.

## Introduction

HFRS is caused by various types of hantaviruses and is a natural epidemic disease, with rodents as the main source of infection^1^. Hantavirus infection is associated with HFRS and hantavirus pulmonary syndrome^2,3^.

The main animal hosts are rodents, followed by cats, pigs, and dogs. *Apodemus agrarius* and *Mus norvegicus* are the main hosts and sources of infection in China^4^. In addition, raising or keeping pet rats can cause Seoul virus infection due to close contact with the infected animal, further increasing the incidence of HFRS^5–8^. The main routes of transmission include contact with rodent feces and other respiratory or digestive tract secretions, as well as contact transmission.

Seoul virus is spread globally, and has been found in mice in the UK, France, Sweden, and Belgium^9^. HFRS is thus prevalent worldwide, with Eurasia ^10^ particularly China, having the highest prevalence. In 2000–2017, Russia reported 131,590 cases of viral HFRS caused by six different hantaviruses^11^. The average annual incidence rates were 6 cases per 100,000 people in western Russia and 0.4 cases per 100,000 people in eastern Russia. From January 2014 to June 2019, nearly 60,000 cases of HFRS were reported in mainland China, with 360 deaths and a case fatality rate of 0.6% (data source, Chinese Centers for Disease Prevention and Control (CDC) http://www.chinacdc.cn/). In China, the areas with the highest prevalence of HFRS include Northeast China, Shanxi, and Shandong Provinces^12^. Tai’an is a prefecture-level city in Shandong Province; however, the incidence of HFRS in Tai’an differs from that of Shandong as a whole. Many studies have reported that HFRS mainly affects farmers^13,14^. It is not clear whether other groups are at a higher risk of disease. In addition to vertical transmission, there have also been a few reports of human-to-human transmission among the general public. Moreover, the differences in the risk according to age and sex are not clear. In addition to being related to hantavirus infection, HFRS is also affected by environmental and meteorological factors^15–21^. Climatic and environmental anomalies directly contribute to the outbreak or expansion of various public health diseases, including HFRS^22^. In Finland, Puumala hantavirus infection was not associated with cardiovascular disease, lung disease, kidney disease, and cancer, but was associated with long-term smoking^23^. Compared with our previous reports on HFRS^24^, the present study aims to provide detailed characteristics of patients with HFRS, provide visual data, and analyze the relative risk of HFRS over a duration of 15 years In this study, we determined the incidence risk of HFRS among different populations in Tai'an, China, as well as its relationship with time and meteorological factors.

## Results

### Epidemic profile

Tai'an is a prefecture-level city under the jurisdiction of the Shandong Province. It is located between 116°02' and 117°59' E and 35°38' and 36°28' N. It is approximately 176.6 km from east to west and 93.5 km from north to south. We can easily find the Tai'an area through the following online mapping software (https://ditu.so.com/?t=map&src=onebox&new=1&dspall=0&k=%E6%B3%B0%E5%AE%89%E5%9C%B0%E5%9B%BE%E7%99%BE%E5%BA%A6&c=&new=1).

From 2005 to 2019, 482 cases of HFRS occurred in the Tai'an area, corresponding to an average annual incidence rate of 0.57/100,000. Nine patients had died, and the case fatality rate of HFRS was 1.87% (95% confidence interval [CI]: 0.66–3.08%). Eight (1.66%) cases were initially misdiagnosed as severe fever with thrombocytopenia syndrome (SFTS).

### Time distribution

**Figure 1:**
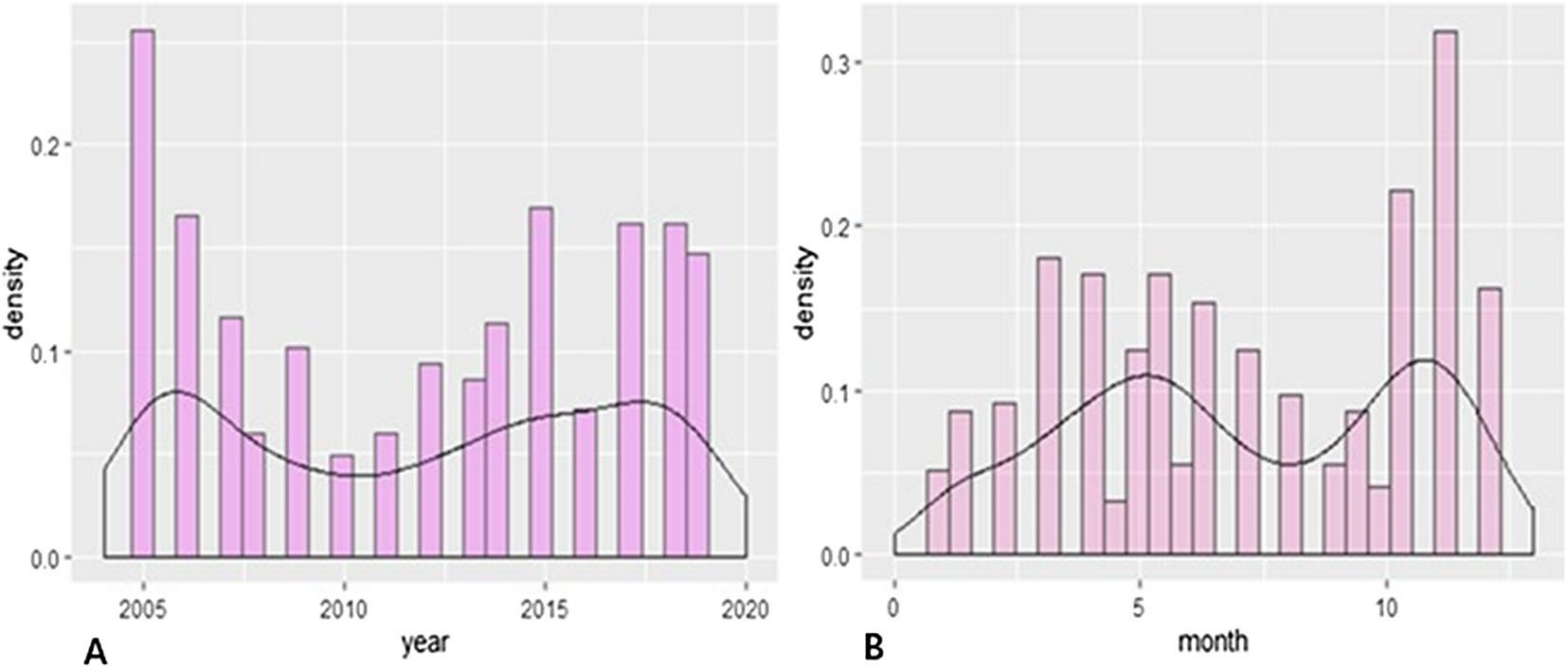
**(A)** Distribution of the annual number of cases from 2005 to 2019. **(B)** Distribution of the monthly number of cases from 2005 to 2019.

Analysis of HFRS cases over 15 years, between 2005 and 2019, showed two incidence peaks: in 2005–2006 and 2017–2019. These peaks accounted for 47.5% of all cases. The highest number of cases (n = 60) occurred in 2005, while the lowest number of cases (n = 13) occurred in 2010 (Fig. 1A).

Exploration of the total number of cases per month over 15 years also showed two HFRS incidence peaks in the Tai'an area: one in the summer months and one in the winter months. The summer peak occurred from March 16 to June 15, with a total of 160 cases over 15 years (33.2%). The winter peak occurred from September 16 to December 15, with a total of 172 cases (35.7%) (Fig. 1B).

Among the nine deaths in 15 years, six occurred during the winter months (November–January).

### Population distribution

#### Sex distribution

Over the 15-year study period, 142 women accounted for 29.5% of all cases, while 340 men accounted for 70.5% of patients. The average annual incidence rate in women was 0.35 per 100,000 population (95% CI: 0.130.57 100,000. The average annual incidence rate in men was 0.81/100,000 people (95% CI: 0.47–1.14/100,000).

#### Occupation distribution

The three occupations with the highest numbers of cases were farmers (74.07%); skilled workers, including teachers, students, cadres, medical staff, and retirees (12.03%); and skilled workers (8.71%). Six patients were medical staff (1.24%).

#### Age distribution

The median patient age was 47 years (range: 3–85), including 29 patients aged < 18 years (6.02%), 97 patients between 19 and 35 years (20.12%), and 296 patients between 36 and 65 years (61.41%). Only 60 patients were aged > 65 years (12.45%). The peak age of onset for HFRS was between 36 and 65 years.

### Relative risks

#### Occupational risk

**Figure 2:**
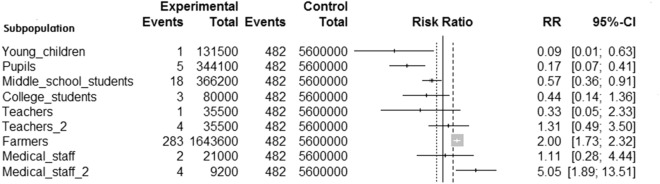
Risk of different populations compared with that of the whole population. Teachers: teachers outside rural areas. Teachers_2: teachers in rural areas. Medical staff: medical staff outside rural areas. Medicalstaff_2: medical staff in rural areas.

Compared with the whole population in the Tai'an area, the risk was significantly lower in young children, primary school students, and middle school students (Fig. 2). The risk of disease among college students, teachers, and non-rural medical staff was moderate, with no significant difference. Farmers (aged 25–64 years) had a higher risk of the disease, with a risk ratio (RR) of 2.00 (95% CI: 1.73–2.32, *p* < 0.05), than others. Rural medical staff had the highest risk of developing the disease, with an RR of 5.05 (95% CI: 1.89–13.51, *p* < 0.05).

#### Age and sex risks

**Figure 3:**
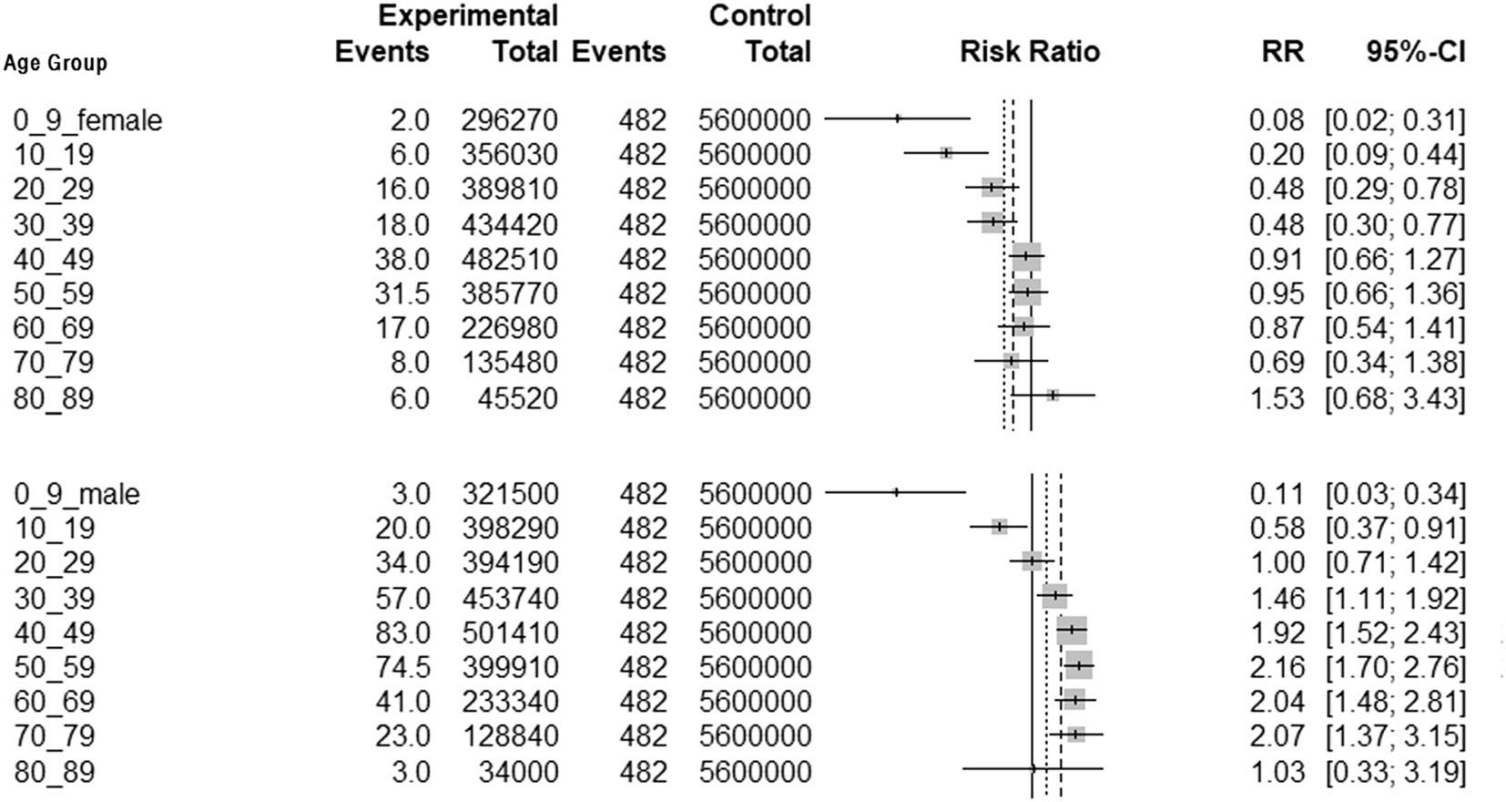
Risks of disease in different age groups compared to the whole population (women on top, men below).

Compared with the control group, women aged 0–39 years had a significantly low risk of onset (Fig. 3). Women aged > 40 years had a moderate risk, with no significant difference. Men aged 0–19 years had a low risk of developing the disease (*p* < 0.05, Fig. 3). However, men aged 30–79 years had a high risk of developing the disease. The risk was the highest for patients aged 50–59 years, with an RR of 2.16 (95% CI: 1.70–2.76, *p* < 0.05). The risk of developing the disease in women aged 10–79 years was lower than that in men (*p* < 0.05, S1 Fig).

### Joinpoint models

**Figure 4:**
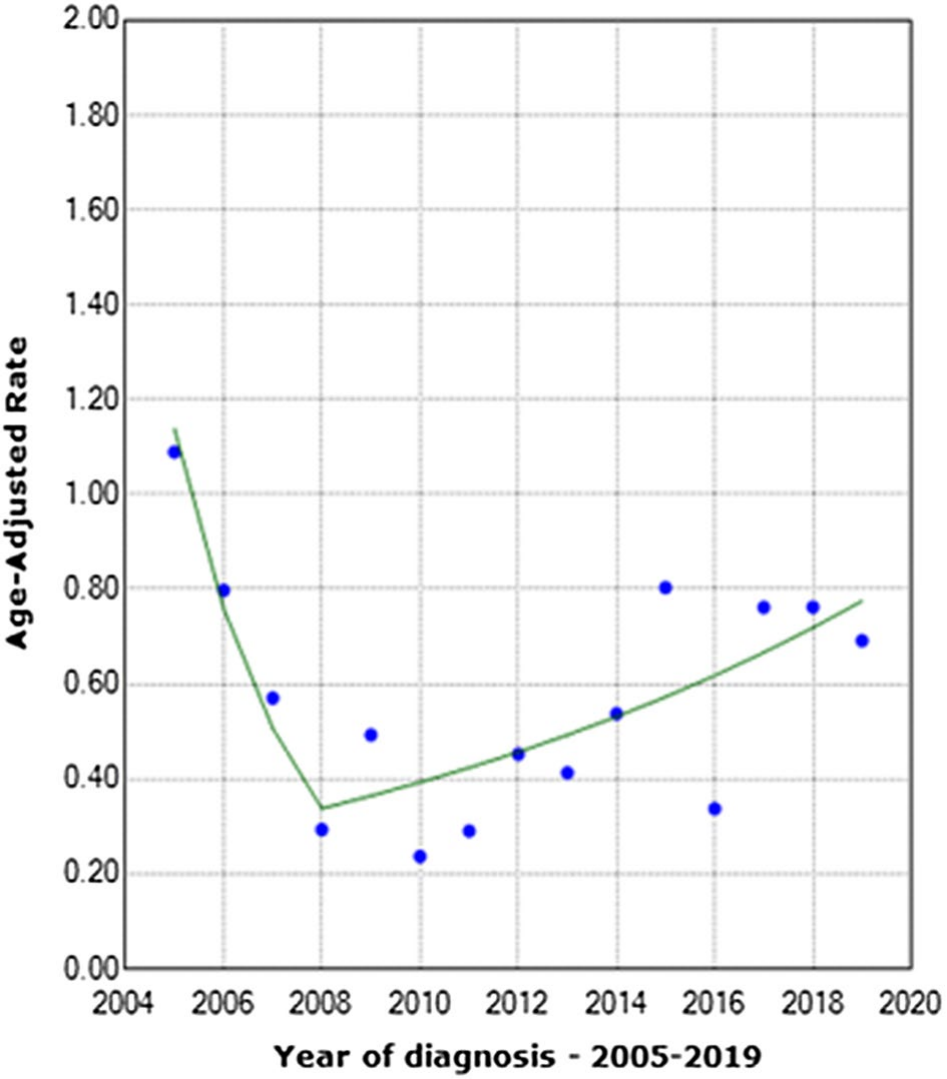
Joinpoint models showing the changes in average annual incidence rates from 2005 to 2019.

The joinpoint models showed that the total number of cases decreased by an average of 33.32% per year from 2005 to 2008 (*p* < 0.05, Fig. 4). From 2008 to 2019, the incidence increased by an average of 7.85% per year (p > 0.05). For women, the incidence increased by an average of 0.93% per year from 2005 to 2019 (p > 0.05, S2 Fig), and for men, this decreased by an average of 38.71% per year from 2005 to 2008 (*p* < 0.05, S3 Fig). However, from 2008 to 2019, the incidence in men increased by an average of 9.24% per year (*p* < 0.05).

### Generalized additive models (GAMs)

**Figure 5:**
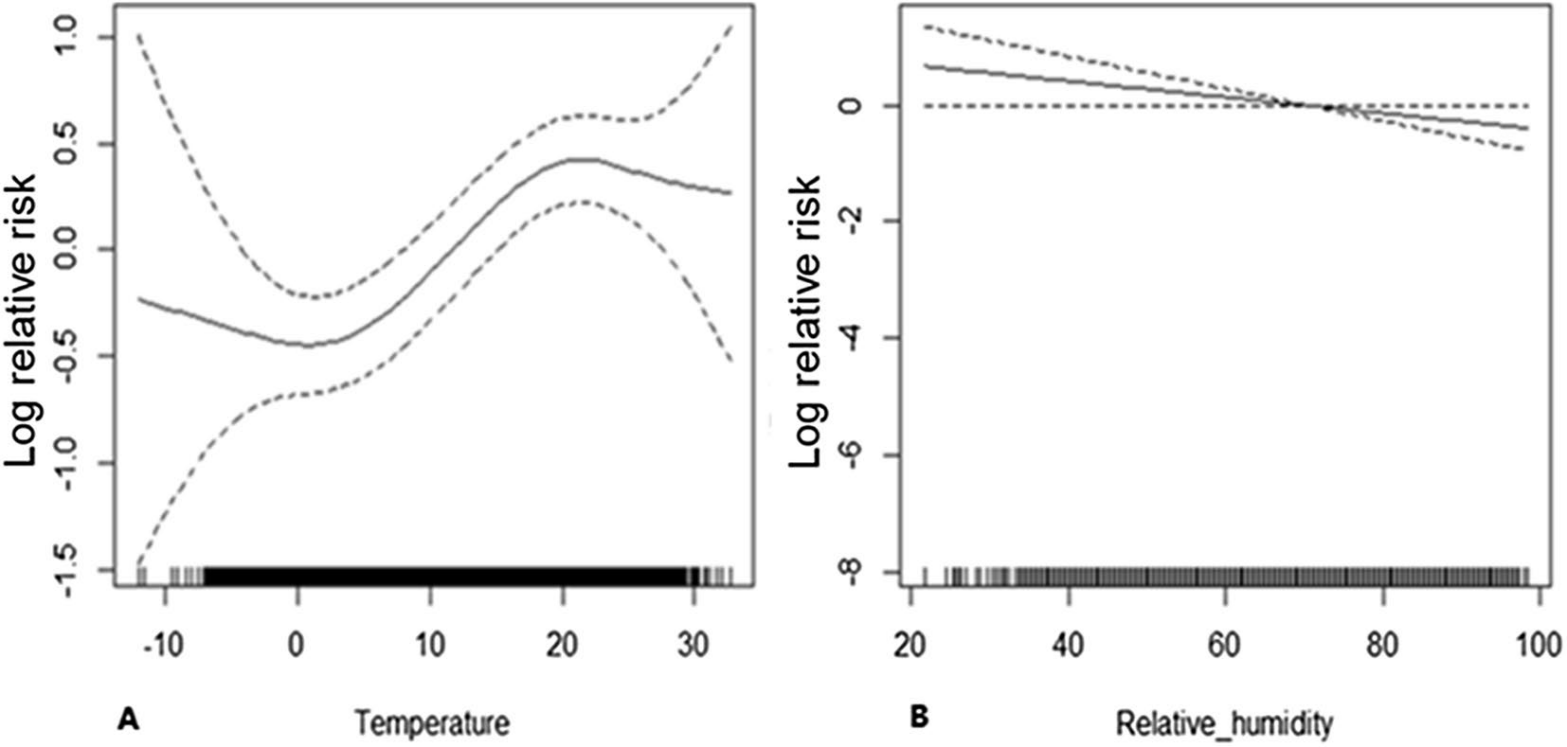
**(A)** Relationship between temperature and disease risk. **(B)** Relationship between relative humidity and disease risk.

We observed a positive correlation between temperature and disease risk from January to June (p < 0.01, Fig. 5A and Supplementary 2), but did not observe significant differences in terms of relative humidity (p = 0.059) or sunshine duration (p = 0.38). Overall, for every 1-unit increase in temperature, the risk of disease increased by 3%.

However, we observed a negative correlation between relative humidity and disease risk (*p* < 0.05, Fig. 5B) from July to December, while temperature (p = 0.17) or sunshine hours (p = 0.24) did not differ significantly. Overall, for every unit increase in relative humidity, the risk of disease decreased by 1%.

### Distributed lag models

We built a bi-dimensional distributed lag nonlinear model (DLNM) using the DLNM package. We found that compared to the reference temperature (20 °C), 1–2 lags had a low risk at low temperatures (< 15 °C) from January to June. However, at high temperatures (> 30 °C), compared to 20 °C, 7–14 lags showed a lower risk (S4 Fig A). From July to December, there was a high risk for 4–10 lags at low temperatures (< 15 °C) compared to that at 20 °C. However, at low temperatures, 12–15 lags had a low risk. At high temperatures, 7–12 lags showed a low risk (S4 Fig B).

From January to June, 1–14 lags showed a low risk of developing the disease at a low relative humidity (< 40%). From July to December, when the relative humidity was low, there was a high risk compared to the control (relative humidity 60%) of 7–12 lags. When the relative humidity was very high, there was a low risk of 7–10 lags.

We also established DLMs for seasonal analysis to calculate the cumulative disease risks. From January to June, at low temperatures, − 5 °C increased to 5 °C, and after 15 lags, the cumulative risk of disease increased (RR = 2.4, 95% CI: 1.48–3.88, *p* < 0.05). At high temperatures, 15 °C increased to 25 °C, and after 15 lags, the cumulative risk was 0.004 (95% CI: 0.001–0.014, *p* < 0.05). In the same period, after 15 lags, when the relative humidity increased from 30 to 40%, the cumulative risk was 0.93 (95% CI: 0.76–1.13, p > 0.05). However, the cumulative risk increased when the relative humidity increased from 80 to 90% (RR = 0.63, 95% CI: 0.08–5.21, p > 0.05).

From July to December, at low temperature, − 5 °C rose to 5 °C, and after 15 lags, the cumulative risk was 1.46 (95% CI: 0.86–2.46, p > 0.05). At high temperatures, 15 °C increased to 25 °C, and after 15 lags, the cumulative risk was 0.004 (95% CI: 0.0001–0.28, *p* < 0.05). In the same period, after 15 lags, when the relative humidity increased from 30 to 40%, the cumulative risk was 0.66 (95% CI: 0.53–0.83, *p* < 0.05, S5 Fig A&B). Under the same conditions, the cumulative risk increased when the relative humidity increased from 80 to 90% (RR = 0.14, 95% CI: 0.05–0.38, *p* < 0.05).

## Discussion

Together with the CDC in Tai'an City, this study conducted a comprehensive analysis of HFRS over 15 years. The average annual incidence of the disease was 0.57/100,000, and 1.66% of the cases were initially misdiagnosed as SFTS. Two incidence peaks were observed in the past 15 years. In terms of months, there were two incidence peaks in the summer and winter. In this study, 70.5% of the patients were men. Regarding occupation, 74.07% of the cases were farmers and migrant workers. Four patients were rural medical staff. Compared to the whole population, the RR of disease for the agricultural population was 2.00, and that of rural medical staff was as high as 5.05. In terms of age, men aged 30–79 years were at a higher risk than others. In terms of sex, the risk of disease in women aged 10–79 years was lower than that in men. Overall, the incidence rate decreased from 2005 to 2008. In the first half of the year, for every unit increase in temperature, the risk of disease increased by 3%. In the second half of the year, for every unit increase in relative humidity, the risk of disease decreased by 1%. From January to June, the temperature increased from − 5 to 5 °C, and the cumulative risk of disease increased after 15 lags (RR = 2.4). However, we found that the cumulative risk was reduced at high temperatures. Specifically, when the temperature increased from 15 to 25 °C, the cumulative risk decreased after 15 lags (RR = 0.004). From July to December, after 15 lags, when the relative humidity increased from 30 to 40%, the cumulative risk was 0.66. The cumulative risk (RR = 0.14) increased when the relative humidity increased from 80 to 90%.

Our research showed that of the patients assessed, middle-aged patients were predominantly affected. Furthermore, most patients were men, and the main occupation was farming, consistent with the findings of previous reports^1,13,14,25^. The risk prediction model showed that cultivated land was the highest risk factor for HFRS^16^. In contrast to the previous literature^26^, the disease showed a bimodal structure in this region throughout the year, rather than a single peak. These double peaks may be related to the living habits of local mice.

To the best of our knowledge, this study is the first to report the relative risks of different groups. Previous studies have suggested that farmers account for the majority of patients, but do not indicate that farmers have the highest risk of disease. Our study found that the risk of disease in farmers was twice that in the entire region. On one hand, farmers have a low income and poor living environment, and they may thus have more opportunities to come into close contact with rats; on the other hand, in agricultural production, farmers have more direct or indirect contact with farm voles, which increases their risk of infection. The RR of infection among medical staff in rural areas was 5.05. However, the risk of infection was not high among medical staff outside rural areas. The income of rural teachers is similar to that of rural doctors, but the risk for rural teachers is not as high as that of rural doctors. Medical workers in rural areas come in contact with many patients with fever of unknown causes or with hidden infections, which are difficult to detect. This may explain why the risk in rural medical staff was higher. In short, from the high risk in rural medical workers, we suspect the possibility of human-to-human transmission, although medical workers may also be infected at home. Because the number of infections was limited, it is worth paying attention to the rural medical staff and statistically analyzing their past diseases over a larger area to put forth a stringent argument. The risk of infection in men aged 30–79 years was higher than that in the whole population and for women in the same age group. Men in this age group have a higher risk of disease, which may be related to their patterns of physical activity and differential susceptibility according to sex.

Previous studies have used different mathematical models to predict HFRS, including the ARIMA model^27^. Our study used four models to fit the data and to predict the incidence of the disease. The joinpoint models showed that the total number of cases decreased by an average of 33.32% per year from 2005 to 2008. This finding may be due to economic development, rural urbanization, or improved living conditions. In recent years, we observed a slight increase in the number of cases. In China, significant positive and negative correlations between the HFRS incidence and urbanization have been reported in the primary (1963–1990) and secondary (1991–2010) stages of urbanization, respectively^28^.

Although previous studies reported a relationship between HFRS and rainfall^29,30^, our analysis at different monitoring points showed wide rainfall variations between the meteorological monitoring points approximately 100 km apart; thus, we believed that the error in including rainfall in the model was too large. Therefore, we do not advocate the use of rainfall for predicting the incidence of the disease. Overall, the GAM showed that the risk increased by 3% for every 1 unit increase in temperature in the first half of the year and decreased by 1% for every 1 unit increase in relative humidity in the second half of the year. The reason may be that with the rise of temperatures in the first half of the year, and June being the summer harvest season, the activities of rats increase, thus increasing the number of cases. Meteorological factors have a lag effect on the disease onset ^26^. From January to June, at low temperatures (− 5 to 5 °C), after 15 lags, the cumulative risk was significantly higher. However, the cumulative risk is reduced at high temperatures. A possible reason is that under low temperatures, immunity of the human body decreases, and the cumulative incidence increases; whereas in the case of high temperatures, the viability of the virus decreases, and the immunity of the human body increases; thus, the cumulative incidence decreases. From July to December, the cumulative risk of 15 lags decreased with an increase in the relative humidity at different stages. In the second half of the year, the number of cases may decrease due to the increase in relative humidity, which will reduce people's farmland working time and the level of mouse activity. This shows that air temperature and relative humidity are important environmental factors affecting HFRS.

### Limitations

As the data source is limited to Tai'an City, the number of cases is relatively small, and the risk of different occupations needs to be further verified using a larger sample of data. On the other hand, for many farmers, due to the gradual decrease in the area of cultivated land, the main occupation is not agriculture, and some farmers may also engage in other work. Thus, there is a certain error in calculating the disease risk of farmers. Wind velocity may also have an effect on the onset of HFRS. The relationship between this factor and HFRS was not analyzed due to the lack of wind velocity data.

## Conclusions

The incidence of HFRS in the Tai’an area of Shandong Province showed two peaks every year. The summer peak occurred from March 16 to June 15, while the winter peak occurred from September 16 to December 15. Compared with the whole population, the RR of disease for farmers was 2.00. Rural medical staff had the highest risk of infection, with an RR as high as 5.05. It is worth exploring the possibility of human-to-human transmission of HFRS among rural medical workers in the future. The risk of infection in men aged 30–79 years was higher than that of women of the same age group and the population as a whole, which may be related to a wide range of activities, greater participation in agricultural production activities, and sex susceptibility of men. With economic development, the incidence of this disease remains low. Temperature and relative humidity were the two main environmental factors (with lagged effects) affecting the incidence of hemorrhagic fever.

## Materials and methods

### Data collection

Patient data were obtained from the Tai’an CDC. Every hospital in the Tai'an area reports cases to the CDC at all levels through the China Information Network System of Disease Prevention and Control. This system does not contain patient treatment information. All cases were clinically confirmed based on the clinical manifestations and treatment response. The clinical diagnosis was based on the common clinical manifestations of hemorrhagic fever, such as fever for several days, headache, low back pain, and oliguria, as well as unresponsiveness to antipyretic treatment, and was sometimes combined with a virological diagnosis. When using the Infectious Disease Reporting System, it is necessary to upload the patient’s medical records within 24 h after clinical diagnosis, such as the patient's name, ID card number, home address, main symptoms, and whether it was a diagnosed case or a suspected case. Meteorological data from 2005 to 2018 were obtained from three meteorological monitoring stations around Tai'an. This study included the averaged data from these three monitoring sites. Population information was obtained from the Tai'an Statistics Bureau. When one variable was assessed, other relevant factors were not specifically addressed. The study was approved by the Ethics Committee of Shandong First Medical University (ethics approval No: 202005004). All patient data were anonymized.

### Methods and models

We calculated the number of cases in each year from 2005 to 2019 and plotted them on a density map. Similarly, we calculated the monthly number of cases and deaths over 15 years. We also plotted the age distribution of patients on a density map.

We calculated the RR for different occupations, age groups, and sexes. RR = number of positive events in the experimental group/number of positive events in the control group. The population base of each subgroup was the average reported in the statistical yearbooks of Tai'an in 2011, 2012, and 2014. Considering the floating population every year, the total population of Tai'an is 5.6 million, of which the agricultural population accounts for approximately 50%. Since one patient in the 50–59 age group did not report their sex, we divided 1 equally between the male and female groups by 0.5. Study design: The total population of the Tai'an area served as the observed population of the control group. The experimental group included a specific group of people, such as farmers and teachers. There were some differences in the working environments between these groups. Setting: Hospitals and medical facilities were spread throughout Tai'an were assessed. The data were obtained from the China Information Network System of Disease Prevention and Control.

We used Joinpoint Trend Analysis (version 4.5) to analyze the annual HFRS trends from 2005 to 2019. The principle of the software can be explained by referring to the following pages (https://surveillance.cancer.gov/joinpoint/). We used the “mgcv” software package (https://cran.r-project.org/web/packages/mgcv/index.html) to fit the generalized additive model (GAM) in which a linear predictor is given by the sum of the smoothing functions of user-specified covariates plus the normal parameter components of the generalized linear model. Detailed explanation of the joinpoint and GAM models are published in our previous study^26^.

We used the "dlnm" package (https://cran.rproject.org/web/packages/dlnm/index.html) to establish a distributed lag model of morbidity and meteorological factors. The package “dlnm” contains functions to specify and interpret the DLM and DLNM models. The first step involved selecting two sets of basic functions for exposure-lag-response correlation modeling. The cross-basis function generates the basic matrix of the exposure reaction and the lag-reaction relation and combines them to form a cross-base cross-basis by a special tensor product. We performed modeling using the “glm” function and forecasting with “crosspred” function. We divided each year into the first and second halves to perform a seasonal analysis. We set the lag to 15, the temperature range to -12–33 °C, and the relative humidity to 20–100%. For a more detailed implementation of DLMs and DLNMs, please refer to the example dlnmTS documentation (https://cran.rproject.org/web/packages/ dlnm/vignettes/ dlnmTS.pdf).

### Statistical analysis

The tests of significance used a Monte Carlo permutation method in the joinpoint trend analysis. Other analyses were mainly performed in the R 3.61 (https://www.r-project.org/) language. All statistical tests with *p* < 0.05 were considered statistically significant.

## Supplementary Information

Supplementary Information.

Supplementary Figures.

## References

[1] Lee SH, Kim WK, No JS, Kim JA, Kim JI, Gu SH (2018). Multiplex PCR-based next-generation sequencing and global diversity of seoul virus in humans and rats. Emerg. Infect. Dis..

[2] Li Y, Cazelles B, Yang G, Laine M, Huang ZX, Cai J, Tan H, Stenseth NC, Tian H (2019). Intrinsic and extrinsic drivers of transmission dynamics of hemorrhagic fever with renal syndrome caused by Seoul hantavirus. PLoS Neglect. Trop. Dis..

[3] de St MA, Ervin E, Schumacher M, Yaglom H, VinHatton E, Melman S, Komatsu K, House J, Peterson D, Buttke D (2017). Exposure characteristics of hantavirus pulmonary syndrome patients, United States, 1993–2015. Emerg. Infect. Dis..

[4] Clement J, LeDuc J, McElhinney L, Reynes J, Van Ranst M, Calisher C (2019). Clinical characteristics of ratborne Seoul Hantavirus disease. Emerg. Infect. Dis..

[5] Kerins JL, Koske SE, Kazmierczak J, Austin C, Gowdy K (2018). Outbreak of Seoul virus among rats and rat owners—United States and Canada, 2017. MMWR Morbidity Mortality Wkly. Rep..

[6] Swanink C, Reimerink J, Gisolf J, de Vries A, Claassen M, Martens L, Waegemaekers T, Rozendaal H, Valkenburgh S, Hoornweg T, Maas M (2018). Autochthonous human case of Seoul virus infection, the Netherlands. Emerg. Infect. Dis..

[7] Fill MM, Mullins H, May AS, Henderson H, Brown SM, Chiang CF, Patel NR, Klena JD, Maurice AD, Knust B, Nichol ST (2017). Notes from the field: Multiple cases of Seoul virus infection in a household with infected pet rats—Tennessee, December 2016-April 2017. MMWR Morbidity Mortality Wkly. Rep..

[8] McElhinney LM, Marston DA, Pounder KC, Goharriz H, Wise EL, Verner-Carlsson J, Jennings D, Johnson N, Civello A, Nunez A (2017). High prevalence of Seoul Hantavirus in a breeding colony of pet rats. Epidemiol. Infect..

[9] Ling J, Verner-Carlsson J, Eriksson P, Plyusnina A, Löhmus M, Järhult JD, van de Goot F, Plyusnin A, Lundkvist Å, Sironen T (2019). Genetic analyses of Seoul hantavirus genome recovered from rats (*Rattus norvegicus*) in the Netherlands unveils diverse routes of spread into Europe. J. Med. Virol..

[10] Reynes JM, Carli D, Bour JB, Boudjeltia S, Dewilde A, Gerbier G, Nussbaumer T, Jacomo V, Rapt MP, Rollin PE, Septfons A (2017). Seoul virus infection in humans, France, 2014–2016. Emerg. Infect. Dis..

[11] Tkachenko EA, Ishmukhametov AA, Dzagurova TK, Bernshtein AD, Morozov VG, Siniugina AA, Kurashova SS, Balkina AS, Tkachenko PE, Kruger DH, Klempa B (2019). Hemorrhagic fever with renal syndrome, Russia. Emerg. Infect. Dis..

[12] Zheng Z, Wang P, Wang Z, Zhang D, Wang X, Zuo S, Li X (2019). The characteristics of current natural foci of hemorrhagic fever with renal syndrome in Shandong Province, China, 2012–2015. PLoS Neglect. Trop. Dis..

[13] Wu H, Wang X, Xue M, Wu C, Lu Q, Ding Z, Zhai Y, Lin J (2018). Spatial-temporal characteristics and the epidemiology of haemorrhagic fever with renal syndrome from 2007 to 2016 in Zhejiang Province, China. Sci. Rep..

[14] Çelebi G, Öztoprak N, Öktem İ, Heyman P, Lundkvist Å, Wahlström M, Köktürk F, Pişkin N (2019). Dynamics of Puumala Hantavirus outbreak in Black Sea Region, Turkey. Zoonoses Public Health.

[15] Tian HY, Stenseth NC (2019). The ecological dynamics of hantavirus diseases: From environmental variability to disease prevention largely based on data from China. Plos Neglect. Trop. Dis..

[16] Xiao H, Tong X, Gao L, Hu S, Tan H, Huang ZY, Zhang G, Yang Q, Li X, Huang R, Tong S (2018). Spatial heterogeneity of hemorrhagic fever with renal syndrome is driven by environmental factors and rodent community composition. PLoS Neglect. Trop. Dis..

[17] Jiang F, Wang L, Wang S, Zhu L, Dong L, Zhang Z, Hao B, Yang F, Liu W, Deng Y, Zhang Y (2017). Meteorological factors affect the epidemiology of hemorrhagic fever with renal syndrome via altering the breeding and hantavirus-carrying states of rodents and mites: A 9 years' longitudinal study. Emerg. Microbes Infect..

[18] Xiao H, Tong X, Huang R, Gao LD, Hu SX, Li YP, Gao HW, Zheng P, Yang HS, Huang ZYX (2018). Landscape and rodent community composition are associated with risk of hemorrhagic fever with renal syndrome in two cities in China, 2006–2013. BMC Infect. Dis..

[19] Drewes S, Turni H, Rosenfeld UM, Obiegala A, Strakova P, Imholt C, Glatthaar E, Dressel K, Pfeffer M, Jacob J (2017). Reservoir-driven heterogeneous distribution of recorded human Puumala virus cases in South-West Germany. Zoonoses Public Health.

[20] Tian HY, Yu PB, Cazelles B, Xu L, Tan H, Yang J, Huang SQ, Xu B, Cai J, Ma CF (2017). Interannual cycles of Hantaan virus outbreaks at the human-animal interface in Central China are controlled by temperature and rainfall. Proc. Natl. Acad. Sci. U.S.A..

[21] Tong MX, Hansen A, Hanson-Easey S, Cameron S, Xiang JJ, Liu QY, Liu XB, Sun YH, Weinstein P, Han GS (2017). Health professionals' perceptions of hemorrhagic fever with renal syndrome and climate change in China. Global Planet. Change.

[22] Anyamba A, Chretien JP, Britch SC, Soebiyanto RP, Small JL, Jepsen R, Forshey BM, Sanchez JL, Smith RD, Harris R (2019). Global disease outbreaks associated with the 2015–2016 El Nino Event. Sci. Rep..

[23] Latronico F, Maki S, Rissanen H, Ollgren J, Lyytikainen O, Vapalahti O, Sane J (2018). Population-based seroprevalence of Puumala Hantavirus in Finland: Smoking as a risk factor. Epidemiol. Infect..

[24] Zhang C, Fu X, Zhang Y, Nie C, Li L, Cao H, Wang J, Wang B, Yi S, Ye Z (2019). Epidemiological and time series analysis of haemorrhagic fever with renal syndrome from 2004 to 2017 in Shandong Province, China. Sci. Rep..

[25] Wang X, Shen W, Qin Y, Ying L, Li H, Lu J, Lu J, Zhang N, Li Z, Zhou W, Tang F (2020). A case-control study on the risk factors for hemorrhagic fever with renal syndrome. BMC Infect. Dis..

[26] Bai XH, Peng C, Jiang T, Hu ZM, Huang DS, Guan P (2019). Distribution of geographical scale, data aggregation unit and period in the correlation analysis between temperature and incidence of HFRS in mainland China: A systematic review of 27 ecological studies. PLoS Neglect. Trop. Dis..

[27] Wang YW, Shen ZZ, Jiang Y (2019). Comparison of autoregressive integrated moving average model and generalised regression neural network model for prediction of haemorrhagic fever with renal syndrome in China: A time-series study. BMJ Open.

[28] Tian H, Hu S, Cazelles B, Chowell G, Gao L, Laine M, Li Y, Yang H, Li Y, Yang Q (2018). Urbanization prolongs hantavirus epidemics in cities. Proc. Natl. Acad. Sci. U.S.A..

[29] Yi L, Xu X, Ge W, Xue H, Li J, Li D, Wang C, Wu H, Liu X, Zheng D, Chen Z (2019). The impact of climate variability on infectious disease transmission in China: Current knowledge and further directions. Environ. Res..

[30] Wei Y, Wang Y, Li X, Qin P, Lu Y, Xu J, Chen S, Li M, Yang Z (2018). Meteorological factors and risk of hemorrhagic fever with renal syndrome in Guangzhou, southern China, 2006–2015. PLoS Neglect. Trop. Dis..

