# Supplementary material for: Epidemiology of hemorrhagic fever with renal syndrome in Tai’an area
Source: Sci Rep. 2021 Jul 5;11:11596. doi: 10.1038/s41598-021-91029-1 (PMC8257732; doi:10.1038/s41598-021-91029-1)
Supplement: Supplementary file 2 — Supplementary Figures. [file 41598_2021_91029_MOESM2_ESM.docx]

**Supplementary materials for “Epidemiology of hemorrhagic fever with renal syndrome in Tai 'an area”**

XiuJuan Bi, Shuying Yi, Aihua Zhang, Zhenghua Zhao, Yunqiang Liu, Chao Zhang, Zhen Ye

**
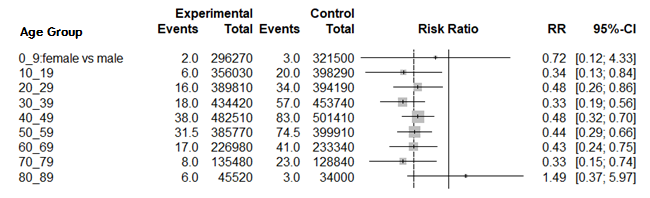
**

S1 Fig. Risks for different age groups according to sex.


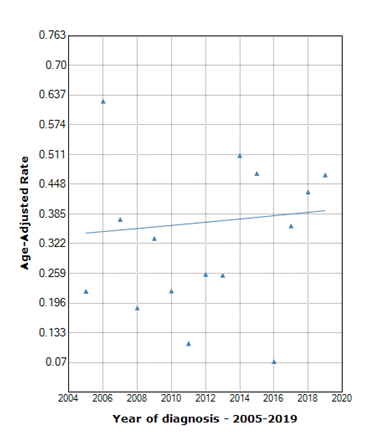


S2 Fig. Joinpoint models showing the changes in average annual incidence rates of females from 2005 to 2019.


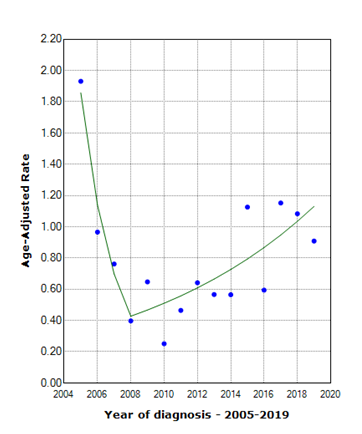


S3 Fig. Joinpoint models showing the changes in average annual incidence rates of males from 2005 to 2019.


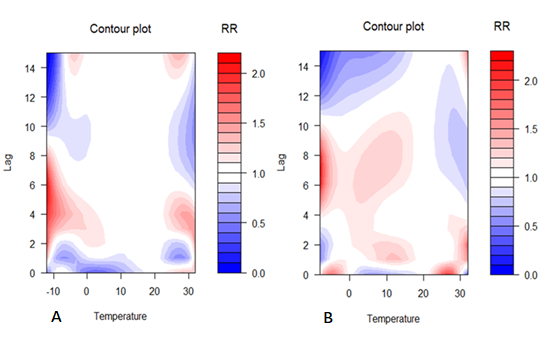


S4 Fig. A. Heat map of the incidence risks and lags at different temperatures from January to June. B. Heat map of the incidence risks and lags at different temperatures from July to December. The darker the red, the higher the risk.


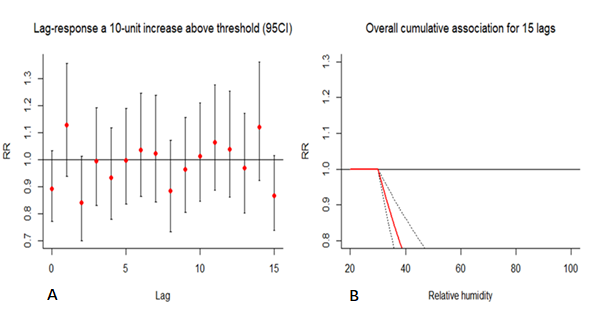


S5 Fig. A. Relative humidity increases by 10 units (30%–40%) and the risks associated with different lags. B. Relative humidity increases by 10 units (30%–40%) after a lag of 15 units on the cumulative risk of disease.
